# Expanding the Fish‐Brain Invitrome With the Senegalese Sole SsB‐1 Cell Line—A Versatile Model for Neurotropic Virus Research

**DOI:** 10.1111/jfd.70197

**Published:** 2026-04-28

**Authors:** Yulema Valero, Alberto Cuesta

**Affiliations:** ^1^ Immunobiology for Aquaculture Group, Department of Cell Biology and Histology, Faculty of Biology University of Murcia Murcia Spain; ^2^ Marine Science Institute (UMUMAR) Murcia Spain

**Keywords:** brain cell line, invitrome, nodavirus, Senegalese sole, SsB‐1, virus

## Abstract

Cell lines are essential tools for studying animal physiology and immunopathology, reducing the use of live animals and supporting the 3Rs principle of reduction, refinement, and replacement. The Senegalese sole ( *Solea senegalensis* ), a relevant species for Spanish aquaculture diversification, lacks established in vitro models. We developed SsB‐1, the first brain‐derived cell line from this species, providing a valuable platform for studying virus–host interactions. SsB‐1 exhibits glial features, stable morphology, and high susceptibility to several betanodavirus (NNV) genotypes, enabling detailed analysis of neurotropism and virulence mechanisms. Its selective permissiveness to NNV and limited replication of infectious pancreatic necrosis virus (IPNV), spring viremia of carp (SVCV), and rhabdovirus eel virus European X (EVEX) highlight its versatility. The ability of some viruses to enter cells without triggering cytopathic or antiviral responses also points to new research avenues for potential reservoir roles in flatfish. Overall, SsB‐1 offers a robust, ethical, and versatile model to advance virological and immunological research in support of sustainable marine aquaculture.

## 1 Introduction

The global aquaculture industry faces increasing pressure to enhance productivity, sustainability, and disease resilience. In Europe, diversification of marine finfish species is a strategic goal to reduce dependence on a few dominant species. The Senegalese sole ( *Solea senegalensis* ) has emerged as a key candidate due to its high commercial value, favourable flesh quality, and strong consumer acceptance across southern Europe (Morais et al. 2016). However, despite decades of interest, its large‐scale production under captivity remains constrained by high mortality during early stages, reproductive challenges, and disease outbreaks (Morais et al. 2016). Recent improvements in broodstock management, selective breeding, and the implementation of recirculating aquaculture systems (RAS) have supported gradual expansion, but disease outbreaks, and particularly viral infections, continue to represent a major bottleneck to further intensification (Bastos‐Almeida et al. 2022) and constrain the species' commercial scalability.

Thus, viruses are the most devastating and difficult to control pathogens, owing to their rapid transmission and lack of effective therapies (Elgendy et al. 2024). Among them, nervous necrosis virus (NNV), responsible for viral encephalopathy and retinopathy (VER), constitutes the only neurotropic virus reported to date in Senegalese sole under farming conditions (Olveira et al. 2009), causing severe mortalities in larvae and juveniles infected with reassortant RGNNV/SJNNV strains (according to RNA1:RNA2 of red grouper─RGNNNV and striped jack─SJNNV parental strains, respectively) (Bandín and Souto 2020; Toffan et al. 2017). As a neurotropic virus, it primarily targets central nervous system (CNS) cells, where it can persist for extended periods in juvenile survivors as well as in asymptomatic adult carriers or resistant species (Toffan et al. 2017; Valero, Olveira, et al. 2021). In contrast, viral haemorrhagic septicaemia virus (VHSV) has only been shown to infect sole under experimental conditions, where it affects both peripheral and central nervous system cells even triggering long period resistant stages (López‐Vázquez et al. 2011; OIE 2009), but not natural infections with confirmed neurotropism have been reported in this species. Whereas these findings highlight the limited diversity of known neurotropic viruses in sole, susceptibility of sole to other RNA viruses with marked CNS tropism—infectious haematopoietic necrosis virus (IHNV), Tilapia lake virus (TiLV), and spring viremia of carp virus (SVCV)—remains unexplored despite their relevance in other teleosts (Dixon 2008; Mugimba et al. 2020; Müller et al. 2015; Wang et al. 2017). Understanding virus‐sole cell interactions is therefore crucial for preventing outbreaks and developing antiviral strategies.

Experimental infections in live fish have historically advanced knowledge of viral pathogenesis but are increasingly constrained by ethical and regulatory considerations, as the principles of the 3Rs (replacement, reduction, refinement) and the increasing limitations of in vivo models. Moreover, mechanistic virus–host studies in virology require tools that enable the investigation of interactions at the cellular level. In this sense, continuous fish cell lines provide a reproducible and ethically preferable alternative to investigate host–virus interactions, screen antiviral compounds, and study innate immune responses. Several brain‐derived cell lines exist from aquaculture‐relevant species, including SaB‐1 from gilthead seabream ( *Sparus aurata* ), DLB‐1 from European sea bass ( *Dicentrarchus labrax* ) or TiB from Nile tilapia ( *Oreochromis niloticus* ) (Morcillo et al. 2017; Ruiz‐Palacios, Esteban, and Cuesta 2020; Wang et al. 2018). Together with other brain‐derived lines such as tongue sole ( *Cynoglossus semilaevis* ) CSAC and Asian sea bass ( *Lates calcarifer* ) BB cell lines, all have proven essential for dissecting viral neurotropism and host immune responses (Chi et al. 2005; Wang et al. 2015), supporting the need for analogous models in European marine aquaculture. However, SSC1, a bone‐derived cell line (Marques et al. 2014), remains the only cell line established from Senegalese sole to date.

Within the invitrome framework, a modular collection of fish cell lines that represents distinct tissue functions (Bols et al. 2017; Revel et al. 2025), brain‐derived lines form the neurotropic module, or the fish‐brain invitrome, which are used to investigate viruses targeting the central nervous system. Rather than a single standardized platform, the invitrome functions as an evolving network for complementary cell‐based models that collectively mirror the tissue and functional diversity across species. Each newly characterized cell line therefore contributes unique biological capabilities to this interconnected system (Bols et al. 2017, Revel et al. 2025). In this study, we expanded the neurotropic module by developing the SsB‐1 cell line from Senegalese sole brain tissue. SsB‐1 expresses glial markers and is susceptible to several viruses, including NNV parental and reassortant strains, SVCV, infectious pancreatic necrosis virus (IPNV), and anguillid viruses such as rhabdovirus eel virus European X (EVEX). However, antiviral responses vary across these infections. Establishing this cell line extends the fish‐brain invitrome to include the first Senegalese sole brain model, offering a valuable platform for investigating NNV and other neuropathogenic agents in a species of growing aquaculture importance.

## 2 Material and Methods

### 2.1 Establishment of the SsB‐1 Cell Line

Senegalese sole ( *Solea senegalensis* ) specimens were generously provided by David García Valcarce (Centro Oceanográfico de Santander, Instituto Español de Oceanografía, CSIC). Brain explants were maintained in culture using flasks (Sarstedt) with L‐15 Leibovitz medium (BioWest), supplemented with 15% foetal bovine serum (FBS, Thermo Fisher Scientific), 4 mM L‐glutamine (Thermo Fisher Scientific), 200 IU/mL penicillin/streptomycin (Thermo Fisher Scientific), and 50 μg/mL amphotericin B (Sigma) at 25°C. Daily monitoring was conducted using an inverted phase‐contrast microscope (Nikon Eclipse TE 2000‐U), and subculturing was performed through standard trypsinization with a 0.25% trypsin solution containing 0.53 mM ethylenediaminetetraacetic acid (EDTA) (Sigma‐Aldrich). Once the culture reached confluence, a retrovirus (SnRV; *Retroviridae* family, *Orthoretrovirinae* genus) was introduced to promote cell immortalization (Morcillo et al. 2017). Culture progression from the explant to the cell line consolidation was routinely followed by an Axio Observer 7 inverted phase contrast microscope (ZEISS) and images acquired with its own image acquisition system (CMOS, 8.3 mp).

The resulting cell line, designated as SsB‐1, has undergone over 90 passages and is routinely maintained in culture with 10% FBS, 2 mM L‐glutamine (Thermo Fisher Scientific), 100 IU/mL penicillin/streptomycin and without amphotericin B. Weekly subculturing is performed after trypsinization, with dilutions ranging from 1:8 to 1:12. Cells are cryopreserved in liquid nitrogen using 10% dimethyl sulfoxide (DMSO) and 30% FBS and recovered through standard thawing procedures. All cell line characterizations were conducted between passages 60 and 80. Mycoplasma contamination was assessed at various passages using the Hoechst 33258 DNA staining method (Chen 1977), consistently yielding negative results (Supporting Information S1).

For scanning electron microscopy (SEM), previously detached cells were washed in 0.01 M phosphate buffer (PBS), fixed in 3% (v/v) glutaraldehyde for 30 min and post‐fixed in 1% OsO_4_ for 1 h. Afterwards, samples were dehydrated in acetone, critical‐point dried, sputter coated with gold and examined with a Jeol 6.100 scanning electron microscope.

### 2.2 Viral Infection

SsB‐1 cells (*n* = 4 wells/virus strain) with a confluence of 70%–80% were incubated in 96‐well plates at 25°C with the following fish virus strains: RGNNV (It/411/96 strain), SJNNV (It/411/97 strain), RGNNV/SJNNV (367.2.2005 strain) and SJNNV/RGNNV (389/I96 strain) reassortants, eel virus European X (EVEX; 690/I88 strain), infectious pancreatic necrosis virus (IPNV; Sp strain), or spring viremia of carp virus (SVCV; 56/70 strain). Cells were infected with multiplicity of infections (MOI) of 0.1 and 1, and incubated at 25°C with L‐15 media supplemented with 2% FBS. The presence of cytopathic effect (CPE) was daily tested until 10 days. Mock‐infected samples were used as controls to distinguish virus‐induced damage from changes caused by the inoculation procedure itself.

### 2.3 Viral Detection and Antiviral Immunity

SsB‐1 cells were seeded in 12‐well plates (Sarsted) overnight and then treated at selected MOIs of 0.1 (EVEX), 1 (IPNV, SVCV, parental or reassortant NNV), or polyinosinic:polycytidylic acid (25 μg mL^−1^ pIC; Sigma‐Aldrich) in supplemented L‐15 medium with 2% SBF. After 24 h, cells were rinsed and stored at −80°C TRIzol Reagent (Thermo Fisher Scientific) for further real‐time or conventional PCR analysis.

### 2.4 Gene Expression Analysis by PCR

Total RNA was extracted from TRIzol Reagent‐frozen samples following the manufacturer's protocol. The RNA's quality and concentration were assessed using a Nanodrop 2000 spectrophotometer (Thermo Fisher Scientific). To eliminate genomic DNA contamination, 1 μg of total RNA was treated with DNase I (Promega). First‐strand cDNA was then synthesized via reverse transcription using Superscript IV (Life Technologies) and random primers.

Real‐time PCR was conducted on a QuantStudio 5 Flex instrument (Applied Biosystems). The reaction conditions included an initial denaturation at 95°C for 10 min, followed by 40 cycles of 95°C for 15 s and 60°C for 1 min, and a final step of 95°C for 15 s, 60°C for 1 min, and 95°C for 15 s. Gene expression levels were normalized using the actin beta (*actb*) expression in each sample and calculated using the 2^−ΔCt^ method (Livak and Schmittgen 2001). Primers were specifically designed using the OligoPerfect software tool (Thermo Fisher Scientific) and are listed in Table 1. A melting curve analysis of amplified products ensured primer specificity in every real‐time PCR run. Negative controls lacking template DNA were always included.

**TABLE 1:** Primer sequences used for conventional and real‐time PCR.

		Coding protein	Gene	Accession number/Reference	Sequence (5′3′)		PCR type	Tm (°C)
Viruses	SnRV	Polymerase	*pol*	SRU26458	F	TGGTACCCATGGATACAGGTACCTCA	Conventional	56
					R	TGTCAGACATGGCCTGTACTTTAGCAGC	One‐round	
	EVEX	Protein L	*evex‐l*	NC_022581.1	F	CTCTAATGTCCTGGGAATTGAGGG	Conventional	58
					R	GATTTTAGATTCTCTTTTGATGACCAACAGGT	Two‐round	
	IPNV	Viral protein 2	*vp2*	Tapia et al. 2015	F	TCCAACTACGAGCTGATCCC	Real‐time	60
					R	GTCCTCTCCTTGTACTCCTC		
	SVCV	Protein N	*svcv.n*	Shao et al. 2016	F	ATCAGGCCGATTATCCTTCCA	Conventional	58
					R	AGATAAGCATTCACATGCTGTAT	One‐round	
	NNV genotypes	Capsid protein	*cp*	D38636; D30814	F	CAACTGACAACGATCACACCTTC	Real‐time	60
					R	CAATCGAACACTCCAGCGACA		
Senegalese sole	Immunity	E3 ubiquitin/ISG15 ligase TRIM25‐like	*isg15*	Labella et al. 2018	F	GGGCCTGAACATACTCCAAA	Real‐time	60
					R	ACCATTGGTGCTTGGTCTTC		
		Double‐stranded RNA‐dependent protein kinase	*pkr*	Labella et al. 2018	F	TAACACGTCGACCCAGTCAA	Real‐time	60
					R	GACAACCTTGACGGCGTAAT		
		IFN regulatory factor 3 variant 1	*irf3x1*	XM_044014056	F	GCCAGCCTTGACTGAGGATT	Real‐time	60
					R	AATCACAGCGTGACTCTGCA		
		Interferon‐induced GTP‐binding protein Mx	*mx*	AY790537	F	TACACGGACAGCCAGTGAAC	Real‐time	60
					R	ATCCTCCTCCTGTGCCATCT		
		Melanoma differentiation‐associated protein 5	*mda5*	XM_044019319	F	CGAGGAGCATTGGGGACTTT	Real‐time	60
					R	GTTGCTGAGATTCAGGCCCT		
		Signal transducer and activator of transcription 1 variant 2	*stat1x2*	XM_044012816	F	AGCTGGGTTGAACACACCAA	Real‐time	60
					R	CTGGTCCTGTTCCCCTGTTC		
	Neuronal markers	Microtubule‐associated protein 2	*map2*	XM_044019143.1	F	CTTTGCACAAGGTGTCCAGC	Real‐time	60
					R	GATAGGTTCTTTGCGCGCAG		
		RNA binding protein fox‐1 homologue 2‐like	*rbfox1like2*	XM_044051619.1	F	GAGGTGGGTGTACAGTGTGG	Real‐time	60
					R	CGGAAGGGGATGTTGGAGAC		
	Glial markers	Glial fibrillary acidic protein	*gfap*	XM_044050464.1	F	ATTTGGGCTCAGATGTGGGG	Real‐time	60
					R	GGACGCGATTCAAAGATGCC		
		Actin binding protein 1A	*coro1a*	XM_044021142.1	F	TCCGTCACGTCTTTGGTCAG	Real‐time	60
					R	GCAGAACCATGAAGGCTCCT		
	Endogenous marker	Actin beta	*actb*	DQ485686	F	GCCCTCTTCCAGCCATCTTT	Real‐time	60
					R	CTGTCAGCAATGCCAGGGTA		

For conventional PCR, one‐ or two‐round reactions were performed. In the first PCR reaction, cDNA and virus‐specific primers (10 μM each; Table 1) were mixed with 10X reaction buffer, 0.5 μL dNTP mix (2.5 mM each), 1.5 mM MgCl_2_ and 0.5 units Taq polymerase (Thermo Fisher Scientific). The cycling reaction was performed in a GeneExplorer Thermal Cycler instrument (Hangzhou Bioer Technology Co Ltd.) using 1 cycle of 95°C for 5 min, 35 cycles of 95°C for 45 s, 56°C–58°C for 45 s, 72°C for 45 s, followed by 1 cycle of 72°C for 10 min. For the two‐round reactions, the first reaction product was diluted 10‐fold and used as template. PCR products were separated on a 1% agarose gel and stained with GelRed Nucleic Acid Gel Stain (Biotium) and visualized under UV light. In all cases, negative or positive (cDNA from viral stocks) were used.

### 2.5 Statistical Analysis and Nomenclature

Results are presented as the mean ± standard error of the mean (SEM). Data were analysed using Student's *t*‐test to determine differences between control and infected groups, with statistical significance set at *p* ≤ 0.05. All analyses were performed with IBM SPSS Statistics 20.

Gene and protein nomenclature in this manuscript adheres to the guidelines established by the Zebrafish Nomenclature Committee (ZNC) for fish genes and proteins, and the HUGO Gene Nomenclature Committee (HGNC) for mammalian genes and proteins.

## 3 Results

### 3.1 Establishment of the *Solea senegalensis* Brain Cell Line SsB‐1

**FIGURE 1:**
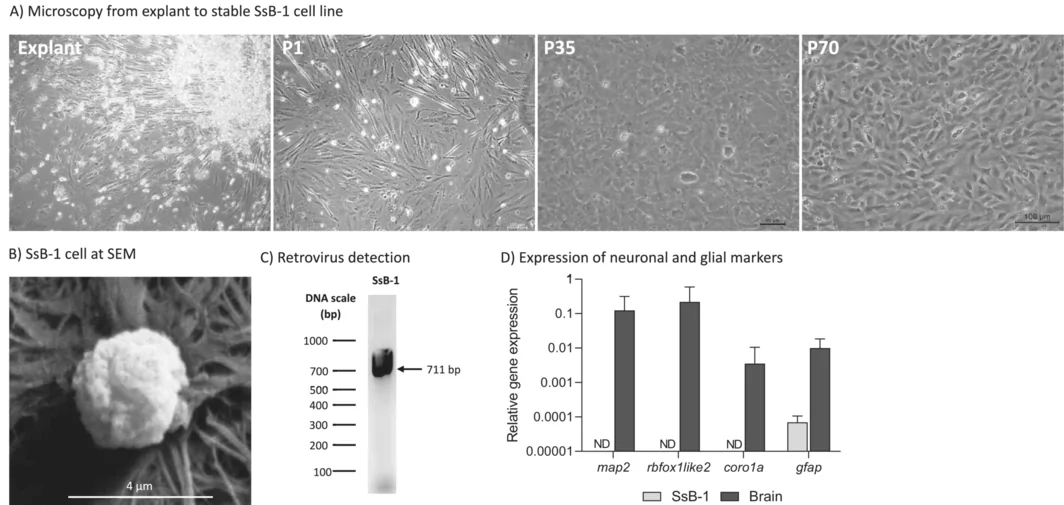
Ssb‐1 cells are neuronal epithelioid‐like cells expressing glial markers. (A) Phase contrast microscopy images from explant and different passages including the stable cell line (P70). (B) Scanning electron microscopy of SsB‐1 cells at stable cell line passage (82). (C) Detection of the retrovirus used for cell immortalization. (D) Expression of neuronal and glial markers by real‐time PCR in SsB‐1 cells and adult sole brain. Data represent the mean relative gene expression ± SEM (*n* = 3). *map2*, microtubule‐associated protein 2; *rbfox1like2*, RNA binding protein fox‐1 homologue 2‐like; *gfap*, glial fibrillary acidic protein; *coro1a*, Actin binding protein 1A.

In this work, we have established the first brain Senegalese sole cell line, denoted as SsB‐1 (Figure 1), using retrovirus immortalization. Within the first few days, cells began to adhere around the explants and proliferated rapidly, eventually reaching confluence (Figure 1A). The initial cultures exhibited heterogeneous morphology, but a fibroblast‐like appearance was predominantly observed (Figure 1A P1); however, they soon adopted a consistent cellular phenotype that has remained stable for over 90 successive passages to date. Established SsB‐1 cells display a predominantly epithelioid morphology, characterized by polygonal shapes, close cell–cell contact, and the formation of a compact monolayer (Figure 1A P35 and P70). The cells appear homogeneous in size and shape, large with well‐defined borders and minimal intercellular spaces, demonstrating a stable and adherent culture (Figure 1A). Upon detachment, the SsB‐1 cells were small and round (3.8 μm) and lacked protrusions, as evidenced by scanning electron microscopy (Figure 1B). Furthermore, cells were successfully recovered after every thawing. Hoechst staining assay for mycoplasma detection always yielded negative results (Supporting Information S1).

Retrovirus was detected by conventional PCR in the established SsB‐1 cell line demonstrating that immortalized cells maintain and support the retrovirus (Figure 1C). To further characterize the cell lineage, only the *gfap* glial marker was detected and lacked those for neuronal cells, *map2* and *rbfox1like2*, all of which were detected in the brain tissue (Figure 1D). These data suggest that SsB‐1 is a glial‐like cell line.

### 3.2 Differential Susceptibility and Permissiveness of SsB‐1 Cells to Viral Infection

**FIGURE 2:**
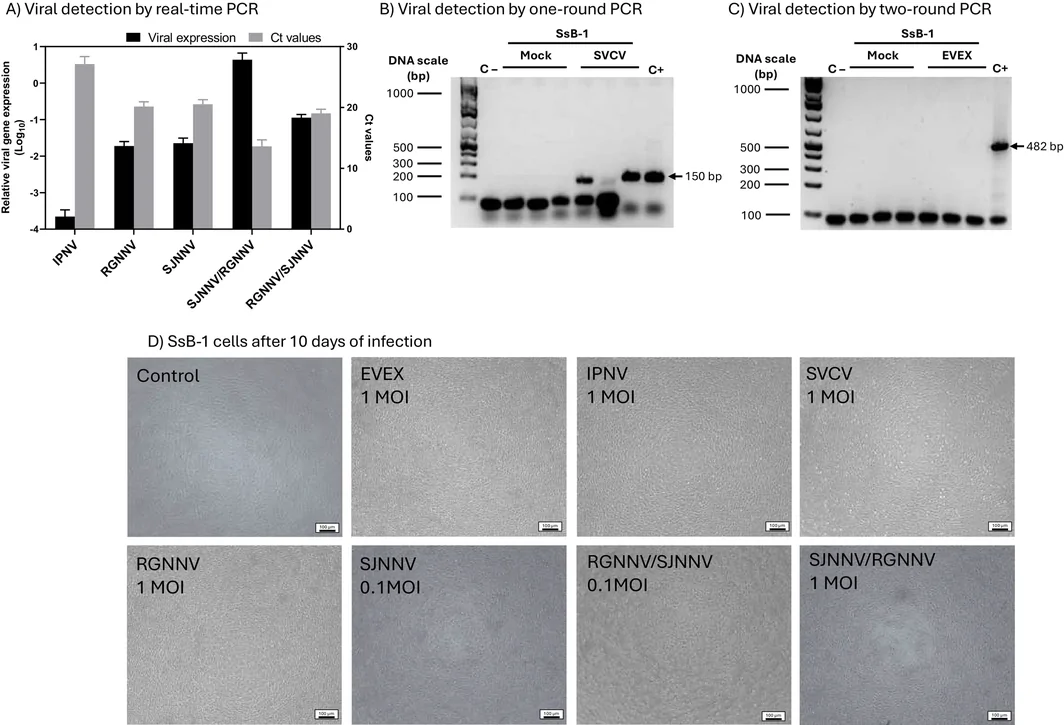
SsB‐1 cells are permissive to several fish viruses. Viral detection and Ct values by real‐time (A), conventional one‐round (B) or two‐round PCR (C) after 1 day of infection. Gene expression was normalized to the house‐keeping gene (*actb*) and presented as mean ± SEM (*n* = 3). C−: Negative control; C+: Positive control. (D) SsB‐1 cell cultures were mock‐ (Control) or virus‐infected (EVEX, IPNV, SVCV, RGNNV, SJNNV, and reassortants RGNNV/SJNNV and SJNNV/RGNNV) and observed after 10 days of infection by phase contrast microscopy. Bars = 100 μm. EVEX, eel virus European X; IPNV, infectious pancreatic necrosis virus; SVCV, spring viremia of carp virus; RGNNV, red‐spotted grouper nervous necrosis virus; SJNNV, striped jack NNV, RGNNV/SJNNV and SJNNV/RGNNV, NNV reassortants.

SsB‐1 cells showed detectable viral RNA for IPNV, SVCV and all NNV genotypes at 24 h post‐infection (hpi), whereas EVEX was not detected by PCR (Figure 2A–C, Table 2). They exhibited varying susceptibility patterns when incubated with different fish viruses at both high and low MOIs, depending on the virus involved (Table 2, Figure 2D). SsB‐1 cells remained unaffected by EVEX, IPNV, SVCV, or RGNNV, showing no apparent CPE at either MOI (Table 2, Figure 2D), which is paralleled to no PCR detection or with high Ct values (Figure 2A–C). On the other hand, infection with SJNNV or NNV reassortants resulted in CPE, demonstrating susceptibility. At low MOI, both RGNNV/SJNNV and SJNNV/RGNNV reassortants triggered partial CPE, characterised by cytoplasmic vacuolation and moderate cell death within the first 24 hpi (Table 2). However, the cells infected with SJNNV/RGNNV recovered rapidly and did not display further CPE during the remaining incubation period while RGNNV/SJNNV, as well as SJNNV, triggered complete CPE at the end of the assay (Table 2). At high MOI, SJNNV and NNV reassortants showed complete CPE after 10 days of infection (Table 2, Figure 2D) with variable kinetics. Thus, SJNNV did very fast as early as 24 hpi, whereas RGNNV/SJNNV did at the 5th day and SJNNV/RGNNV after 10 days. Therefore, SsB‐1 cell line might be a good model to study and understand the susceptibility to SJNNV and NNV reassortants.

**TABLE 2:** Susceptibility of SsB‐1 cells to different fish viruses after 10 days of infection.

Virus	MOI	Incubation period		
		1 day	5 days	10 days
EVEX	0.1	ns	ns	ns
	1	ns	ns	ns
IPNV	0.1	ns	ns	ns
	1	ns	ns	ns
SVCV	0.1	ns	ns	ns
	1	ns	ns	ns
RGNNV	0.1	ns	ns	ns
	1	ns	ns	ns
SJNNV	0.1	ns	Complete CPE	Complete CPE
	1	Complete CPE	Complete CPE	Complete CPE
RGNNV/SJNNV	0.1	Partial CPE	Complete CPE	Complete CPE
	1	Partial CPE	Complete CPE	Complete CPE
SJNNV/RGNNV	0.1	Partial CPE	ns	ns
	1	Partial CPE	Partial CPE	Complete CPE

Abbreviations: CPE, cytopathic effect; EVEX, eel virus European X; IPNV, infectious pancreatic necrosis virus; MOI, multiplicity of infection; ns, no symptoms; RGNNV, red‐spotted grouper nervous necrosis virus; RGNNV/SJNNV, NNV reassortants; SJNNV, striped jack NNV; SJNNV/RGNNV, NNV reassortants; SVCV, spring viremia of carp virus.

### 3.3 Gene Expression Profiles of Type I IFN Markers Following SsB‐1 Viral Infection

**FIGURE 3:**
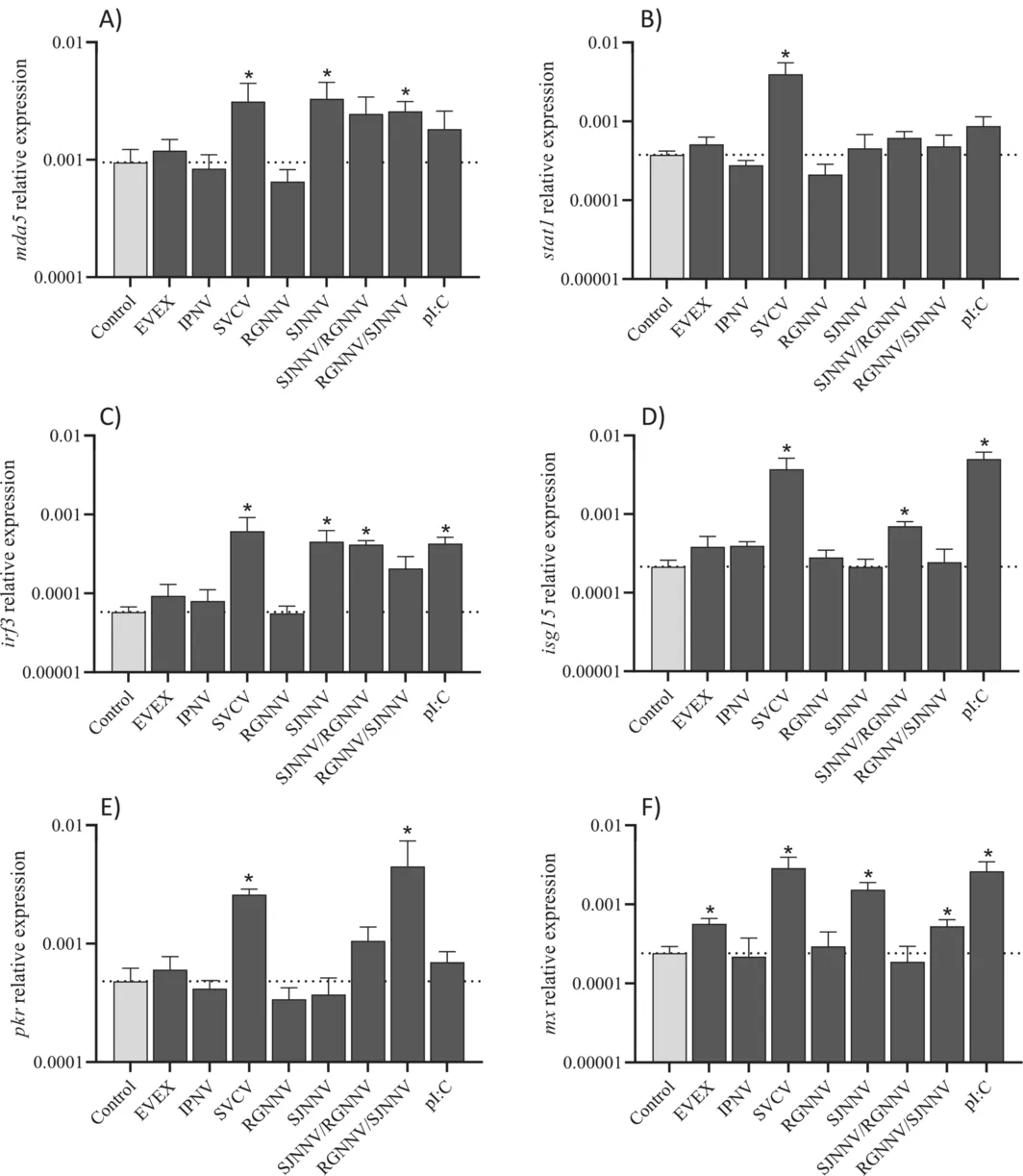
Viral infection of SsB‐1 cells induces differential expression of antiviral genes. Relative expression of (A) *mda5*, (B) *stat1*, (C) *irf3*, (D) *isg15*, (E) *pkr* and (F) *mx* genes in SsB‐1 cells after 1 day of infection with different viruses. Cells incubated with polyinosinic:Polycytidylic acid (25 μg mL^−1^; pIC) were used as positive control. Data represent the mean ± SEM (*n* = 3). Asterisks denote differences between mock (Control) and infected groups (*p* < 0.05). EVEX, eel virus European X; IPNV, infectious pancreatic necrosis virus; SVCV, spring viremia of carp virus; RGNNV, red‐spotted grouper nervous necrosis virus; SJNNV, striped jack NNV, RGNNV/SJNNV and SJNNV/RGNNV, NNV reassortants.

At 24 hpi, SsB‐1 infection with EVE, IPNV, or RGNNV failed to regulate the transcription of type I interferon (IFN) genes whilst the viral mimic pI:C upregulated expression of *irf3*, *isg15*, and *mx* genes (Figure 3). In sharp contrast, SVCV infection induced the expression of all analysed genes (Figure 3). However, SJNNV only induced the expression of *mda5, irf3*, and *mx* genes; RGNNV/SJNNV did for *mda5*, *pkr*, and *mx* whilst SJNNV/RGNNV only up‐regulated to *irf3* and *isg15* genes.

## 4 Discussion

In Mediterranean aquaculture, diversification species such as the Senegalese sole still lack established cell lines (Morais et al. 2016), and no brain‐derived model has been described to date. This gap remains critical given the high susceptibility of this species to neurotropic viruses causing major production losses (Bandín and Souto 2020; Toffan et al. 2017). Fish cell lines are valuable tools for studying physiology, virology, and toxicology, offering broader thermal tolerance, easier maintenance, and greater reproducibility than mammalian systems (Kumar et al. 2024). Here, we report the first brain‐derived cell line from Senegalese sole, SsB‐1, established from brain explants and immortalized by retroviral transduction. SsB‐1 cells display epithelioid‐like morphology, forming polygonal, tightly adherent monolayers while expressing glial markers. Although fish brain‐derived lines with glial features typically shift from epithelioid to elongated or stellate morphologies during subculture (Morcillo et al. 2017; Ruiz‐Palacios, Esteban, and Cuesta 2020; Ruiz‐Palacios, Almeida, et al. 2020; Wang et al. 2015), SsB‐1 has maintained a stable polygonal organization for over 90 passages, without fibroblast‐like subpopulations. Similar morphologies occur in mammalian astrocytes (Souza et al. 2013; Watson et al. 2017), supporting its glial origin despite its epithelioid appearance in vitro. Among flatfish, brain‐derived cell lines have mainly been developed in Asian aquaculture species, including Japanese flounder ( *Paralichthys olivaceus* ; JFB, POBC, POB1, POB2, POBh and POBf), turbot ( *Scophthalmus maximus* ; SMB) and half‐smooth tongue sole ( *Cynoglossus semilaevis* ; CSAC), the latter of astroglial origin (Gao et al. 2020; Sun et al. 2023; Zheng et al. 2015; Wang et al. 2015). In contrast, Senegalese sole represents the first flatfish of major Mediterranean relevance for which a brain‐derived cell line has been established. Currently, the fish brain invitrome comprises around 10 publicly available lines and several others held by research laboratories, mostly from Asian species (Kumar et al. 2024), emphasizing the need for equivalent resources from Mediterranean species.

The SsB‐1 cell line demonstrated high susceptibility to certain NNV genotypes, showing CPE consistent with productive viral replication across multiple genotypes. SJNNV and the reassortants SJNNV/RGNNV and RGNNV/SJNNV successfully infected the cells, with viral RNA detected as early as 24 hpi concomitant with low Ct values, while type I interferon‐related responses remained weakly induced. Indeed, *mx* gene up‐regulation after SJNNV or RGNNV/SJNNV genotypes was not enough to clear the virus. Among them, SJNNV induced the most pronounced CPE, causing complete cell lysis within 1 day at highest MOI. RGNNV/SJNNV produced partial CPE after 1 day and complete lysis by 5 days post‐infection, whereas SJNNV/RGNNV led to a slower progression, reaching total CPE only at the end of the assay. Infections at lower MOIs followed the same trend but with minor effects. Comparable permissiveness patterns have been described in other fish brain‐derived cell lines, such as SaB‐1 and DLB‐1, and the NNV‐reference cell line E‐11; from gilthead seabream, European sea bass and striped snakehead (*Channa striatus*), respectively, which are also permissive to both parental and both reassortant strains, though with species‐specific differences in viral replication kinetics and CPE intensity (Cuesta et al. 2025; Morcillo et al. 2017; Ruiz‐Palacios, Esteban, and Cuesta 2020; Valero, López‐Vázquez, et al. 2021). Comparable to our results, FuB‐1 brain cells from mummichog ( *Fundulus heteroclitus* ) were not permissive to RGNNV in vitro, likely due to the up‐regulation of antiviral genes such as *mx* and *hsp70*, which may have limited viral propagation and prevented cell lysis (Ruiz‐Palacios, Almeida, et al. 2020), while SsB‐1 did not trigger any antiviral response at the transcriptional level. Similarly, GB, GF‐1 and ASBB brain cell lines from yellow grouper ( *Epinephelus awoara* ), orange‐spotted grouper ( *Epinephelus coioides* ) and Asian sea bass ( *Lates calcarifer* ), respectively, showed susceptibility to NNV isolates, supporting the suitability of glial‐derived fish cells as models for NNV replication (Hasoon et al. 2011; Lai et al. 2001; Valero, López‐Vázquez, et al. 2021). However, the rapid and extensive CPE observed in SsB‐1 following infection with SJNNV and reassortant strains indicates higher permissiveness compared to RGNNV, which showed reduced permissiveness. In particular, reassortants expressing the SJNNV capsid (RNA2) may exhibit enhanced cell entry or tropism, whereas RGNNV may display reduced compatibility with SsB‐1 cells at the level of entry or replication. Consistent with this, in vivo studies showed that Senegalese sole is susceptible to both parental genotypes and reassortants, although virulence depends on the exposure route and viral genotype (Souto et al. 2015; Souto, Olveira, Alonso, et al. 2018). Intramuscular infection revealed higher virulence for reassortants expressing the SJNNV capsid, whereas bath exposure resulted in early brain infection with decreasing virulence over time, in contrast to RGNNV, which induced delayed but higher cumulative mortality (Souto et al. 2015; Souto, Olveira, Alonso, et al. 2018). These observations differ from the in vitro scenario, where SsB‐1 appears more susceptible to SJNNV genotype than to RGNNV, which is able to enter into the cells not triggering neither CPE nor antiviral responses. The greater virulence of the RGNNV/SJNNV reassortant in juvenile soles could reflect a functional interaction between the RGNNV‐type RNA1, possibly conferring enhanced replication or immune modulation (Labella et al. 2018), and the SJNNV‐type capsid, better adapted for host cell entry (Souto, Olveira, Alonso, et al. 2018). As a fact, RNA1 may influence replication efficiency and host interaction, while RNA2 (capsid) primarily determines cell entry and tropism (Chen et al. 2015; Olveira et al. 2009; Souto, Olveira, García‐Rosado, et al. 2018; Valero, López‐Vázquez, et al. 2021). This combination may provide an advantage under in vivo conditions that is not evident in vitro, where host complexity and many immune factors are absent (Gémez‐Mata et al. 2021).

Other viruses tested did not trigger visible CPE within 10 days post‐infection. As RGNNV, IPNV was able to enter into SsB‐1 cells, as confirmed by viral detection 24 hpi, despite the absence of cytopathic manifestations or immune response. In contrast, SVCV was detected in infected cultures and induced strong antiviral responses, although the absence of CPE prevents confirmation of productive replication. Interestingly, EVEX was not detected in infected cultures, which may be associated with the observed resistance. Currently, information on the susceptibility of flatfish to these viruses remains scarce. IPNV has only been detected at very low levels in turbot, in cases of co‐infection with RGNNV (Olveira et al. 2013). In contrast, there is no evidence of EVEX or SVCV infection, either acute or persistent, in flatfish species, likely due to the restricted host range of these viruses. However, the absence of large‐scale sequencing studies makes it impossible to fully exclude the possibility that flatfish could act as asymptomatic reservoirs. The ability of these viruses to infect SsB‐1 cells, as indicated by viral detection, suggests that Senegalese sole possesses the cellular mechanisms required for viral entry and potentially for natural infection. Further research is therefore needed to determine whether this species could serve as a reservoir for such viruses in aquaculture environments. These results do not allow discrimination between viral entry and productive replication for viruses that did not induce CPE.

## 5 Conclusion

In summary, the SsB‐1 cell line represents the first brain‐derived model established from Senegalese sole, a species of growing importance in Mediterranean aquaculture. Its glial origin, morphological stability, and high permissiveness to several NNV genotypes make it particularly useful for elucidating mechanisms underlying neurotropism and virulence in nodavirus infections. The differential susceptibility observed among NNV genotypes, together with the limited replication of other viruses such as IPNV, SVCV, and EVEX, highlights both the selectivity and versatility of this cell line as a model system. Moreover, the ability of certain viruses to infect SsB‐1 cells without causing cytopathic effects or triggering antiviral responses opens new perspectives for exploring the potential of flatfish species as viral reservoirs. Overall, SsB‐1 establishes a robust platform for future investigations into virus–host interactions, antiviral responses, and pathogen control strategies, contributing to the development of sustainable and resilient Mediterranean aquaculture.

## Author Contributions

**Yulema Valero:** conceptualization, methodology, investigation, writing – original draft, writing – review and editing, software, data curation. **Alberto Cuesta:** conceptualization, investigation, funding acquisition, writing – review and editing, methodology, project administration, resources.

## Funding

This work was supported by Agencia Estatal de Investigación, PID2019‐105522 GBI00, PID2022‐139492NB‐I00, IJC2020‐042733‐I; Fundación Séneca, 23025/GERM/25.

## Conflicts of Interest

The authors declare no conflicts of interest.

## Supporting information


**Supporting Information: S1** Test for Mycoplasma in SsB‐1 cultured cells at stable passage (P75). (A) Phase contrast. (B) Hoechst staining. (C) Merged. Bar = 50 μm.

## Data Availability

The data that support the findings of this study are available from the corresponding author upon reasonable request.
